# Audit on resuscitation equipment in Carseview Centre (NHS Tayside)

**DOI:** 10.1192/bjo.2021.148

**Published:** 2021-06-18

**Authors:** Thomas Leung, Lina Zariddin, Emma Megoran

**Affiliations:** NHS Tayside

## Abstract

**Aims:**

Psychiatric hospitals are well equipped to manage patients with complex psychiatric needs, however due to their community setting when a rare medical emergency occurs it is not unusual for a small delay whilst staff search for equipment on the ward or even go to other wards for equipment. The aim of this audit is to ensure that our psychiatric wards in Carseview Centre are well equipped to respond to patients becoming medically unwell and put our nurses and doctors in a position to safely stabilise the patient until furthur help arrives.

**Method:**

We collected data from 3 inpatient adult wards, 1 intensive psychiatric care unit and 1 learning disability unit and compared their resuscitation trolley equipment with local NHS Tayside Emergency Equipment Protocol in January 2020. Following data collection we fed back to the wards about our results and discussions were held between doctors, charge nurses, pharmacists and resuscitation officers to determine whether missing equipment were neccesary in the community setting and to see if there were updates that required for our local protocol to better reflect current practices as it had not been reviewed since 2012. Following multiple meetings we amended our local protocol to better reflect what was . A list of recommendations was also made to improve patient safety.

We then collected data again in January 2021

**Result:**

Folloing our first data collection we found that the resuscitation trolleys tended to not have ligature packs and masks were generally not by the oxygen cylinders. Hypoglycaemic dextro-tablets were also not readily available. The Learning disability units also did not have an emergency resuscitation trolley.

Following our discussions and amendment of the protocol this was finalised in November 2020 and was dissemindated towards the wards and we waited 2 months for the changes to take effects and recollected our data. There continued to be equipment that was incomplete/missing on each individual ward, but none that were consistent throughout the whole hospital site. All the recommendations that were made for the 1st data collection had been done.

**Conclusion:**

Overall we felt that the emergency trolleys were better equipped in line with the updated protocol compared to the previous audit cycle. The overall pattern of missing equipment was inconsistent and the recommendation was for staff to copmlete checks to address missing/incomplete items when found. Our local protocol also recommends that all ward should stock ‘additional items’ (nebuliser masks and non-rebreather masks), which majority had however were difficult to locate, which could delay patient care.

We will continue to repeat data collection cycles and feedback to our wards to ensure patient safety is not compromised.

